# Involvement of the Notch pathway in terminal astrocytic differentiation: role of PKA

**DOI:** 10.1042/AN20130023

**Published:** 2013-12-23

**Authors:** Carla Angulo-Rojo, Rebeca Manning-Cela, Adán Aguirre, Arturo Ortega, Esther López-Bayghen

**Affiliations:** *Departamento de Genética y Biología Molecular, Centro de Investigación y de Estudios Avanzados del IPN, México; †Departamento de Biomedicina Molecular, Centro de Investigación y de Estudios Avanzados del IPN, México; ‡Department of Pharmacological Sciences, State University of New York at Stony Brook, NY, U.S.A.

**Keywords:** astrocytic differentiation, cAMP signaling, glia cell, glial transcriptional control, Notch1, protein kinase A (PKA), ADAM, a disintegrin and metalloprotease, CAT, chloramphenicol acetyltransferase, CNS, central nervous system, CRE, cAMP-response element, CREB, cAMP-responsive element binding protein, dbcAMP, dibutyryl-cAMP, DMEM, Dulbecco’s modified Eagle’s medium, GAPDH, glyceraldehyde-3-phosphate dehydrogenase, GFAP, glial fibrillary acid protein, IL-6, interleukin-6, JAK, Janus kinase, NICD, Notch1 intracellular domain, NPC, neuronal progenitor cell, NSC, neural stem cell, PAC1, PACAP (pituitary adenylate cyclase-activating polypeptide) ligand/type 1 receptor, PACAP, pituitary adenylate cyclase-activating polypeptide, PKA, protein kinase A, RBPJκ, recombinant binding protein for immunoglobulin κ region, RT–qPCR, reverse transcription–quantitative PCR, STAT, signal transducer and activator of transcription

## Abstract

The Notch pathway is a highly conserved signaling system essential for modulating neurogenesis and promoting astrogenesis. Similarly, the cAMP signaling cascade can promote astrocytic commitment in several cell culture models, such as the C6 glioma cell line. These cells have the capacity to differentiate into oligodendrocytes or astrocytes, characteristics that allow their use as a glial progenitor model. In this context, we explore here the plausible involvement of cAMP in Notch-dependent signal transactions. The exposure of C6 cells to a non-hydrolysable cAMP analogue resulted in a sustained augmentation of Notch activity, as detected by nuclear translocation of its intracellular domain portion (NICD) and transcriptional activity. The cAMP effect is mediated through the activation of the γ-secretase complex, responsible for Notch cleavage and is sensitive to inhibitors of the cAMP-dependent protein kinase, PKA. As expected, Notch cleavage and nuclear translocation resulted in the up-regulation of the mRNA levels of one of its target genes, the transcription factor *Hair and enhancer of split 5.* Moreover, the glutamate uptake activity, as well as the expression of astrocytic markers such as glial fibrillary acidic protein, S100β protein and GLAST was also enhanced in cAMP-exposed cells. Our results clearly suggest that during the process of C6 astrocytic differentiation, cAMP activates the PKA/γ-secretase/NICD/RBPJκ pathway and Notch1 expression, leading to transcriptional activation of the genes responsible for glial progenitor cell fate decision.

## INTRODUCTION

Astrocytes are generated during CNS (central nervous system) embryonic development from neuronal stem cells and glial progenitors (reviewed in Kriegstein and Alvarez-Buylla, 2009). Astrocyte differentiation is regulated by different extracellular stimuli. Among these, the BMP (bone morphogenic protein), IL-6 (interleukin-6) and neuropeptide PAC1 [PACAP (pituitary adenylate cyclase-activating polypeptide) ligand/type 1 receptor] systems have been characterized (Adachi et al., 2005; Nakamachi et al., 2011).

During development, alterations in the ratio of neurons to glia may severely perturb neuronal function. The seminal observation that neurons are generated prior to glia, suggests that newly generated neurons signal to NPCs (neuronal progenitor cells) to regulate their cellular fate (Temple, 2001). The molecular basis of this signaling has not been completely described, although an interplay between the extracellular cues and intracellular mechanisms in which cell–cell interactions occurs is expected. In fact, embryonic NPCs generate neurons only when they are cultured on top of embryonic cortical slices, in contrast with an enhanced gliogenesis if cells are plated upon adult cortical slices, clearly suggesting that secreted NPC factors regulate the switch from neurogenesis to astrocyte production (Morrow et al., 2001). More recently, the involvement of members of the IL-6 cytokine family has been described as promoters of astrocytic differentiation. Specifically, CT-1 (cardiotropin-1) induces glia commitment in mouse NPCs via the JAK (Janus kinase)/STAT (signal transducer and activator of transcription) pathway (Barnabe-Heider et al., 2005).

Notch1 is a member of a conserved family of transmembrane receptors that participate in cell fate decision during development. Notch family members, are activated by the Delta-like and Jagged ligands. Upon ligand binding, Notch1 undergoes a concerted action of the proteolytic enzymes ADAM (a disintegrin and metalloprotease)/TACE [TNF (tumor necrosis factor)-converting enzyme]/γ-secretase that ultimately release the NICD (Notch1 intracellular domain). The NICD is translocated into the nucleus transforming the CBF1 [RBPJκ (recombinant binding protein for immunoglobulin κ region)]/Su(H)/LAG1(CSL) repressor complex into an activator that induces the transcription of a variety of genes. These include the family of transcription factors *Hes* and *Hey*, known to play a critical role in pro-neural gene repression (Kopan and Ilagan, 2009). In this context, a role for Notch signaling in the NPC-mediated switch to gliogenesis has been suggested (Namihira et al., 2009). NICD up-regulates NFI (nuclear factor I), a member of the CCAAT box element-binding family of transcription factors. Several lines of evidence indicate that active Notch signaling blocks neurogenesis while promoting astrogenesis. Whether Notch is a permissive or an instructive pathway is not clear at this moment (Stockhausen et al., 2010). The cAMP signaling cascade has long been known to be critically involved in transcriptional regulation. The CREB (cAMP-responsive element binding protein) is one of the most studied transcription factors that participate in CNS differentiation (Merz et al., 2011). PACAP, as noted above, is a known glial promoting agent that exerts its activity through G-protein-coupled receptors, leading to an increase in cAMP levels and resulting in an augmentation of the expression of astrocytic genes, such as the intermediate filament GFAP (glial fibrillary acid protein) (Tatsuno et al., 1996). Nestin, another intermediate filament, is transiently expressed in neuroepithelial stem cells of the developing CNS and has been regarded as a useful marker for glial developing cells due to its down-regulation correlating with differentiation into astrocytes, oligodendrocytes or neurons (Lendahl et al., 1990; Hendrickson et al., 2011).

The glioblastoma-derived C6 cell line is morphologically similar to glial precursors and expresses low GFAP levels (reviewed in Grobben et al., 2002). The treatment of these cells with dbcAMP (dibutyryl-cAMP) results in a change in their morphology and the subsequent expression of the astrocytic markers S100β and GFAP, and an increase in [^3^H]D-aspartate uptake activity (Segovia et al., 1994; Yoshimura et al., 1996; Takanaga et al., 2004).

All these observations prompted us to analyze the plausible cAMP/Notch1 interplay during astrocytic differentiation. To this end, C6 cells were differentiated by dbcAMP treatment, and the expression of GFAP, S100β and the Na^+^-dependent glutamate/aspartate transporter (GLAST) was investigated. We were able to establish that cAMP effects are mediated through PKA (protein kinase A) and that the NICD/RBPJκ pathway regulates GFAP, S100β, Notch1 and GLAST expression, supporting the idea that Notch1 acts as a permissive pathway in gliogenesis.

## MATERIALS AND METHODS

### Reagents

dbcAMP, H89 and Forskolin were all obtained from Sigma–Aldrich. InSolution™ γ-secretase inhibitor IX was obtained from Calbiochem.

The antibodies purchased from Santa Cruz Biotechnology were: goat anti-GFAP (catalogue number SC-6170), goat anti-Hes5 (catalogue number SC-13859), goat anti-Notch1 (catalogue number SC-6015), rabbit anti-lamin A/C (catalogue number SC-20681) and rabbit anti-calnexin (catalogue number SC-11397). Rabbit anti-cleaved Notch 1 (Val^1744^; #4147) and rabbit anti-PEN-2 (#5451) were obtained from Cell Signaling Technology. Mouse anti-Notch1 antibody (for Western blot analysis, directed against the extracellular portion, catalogue number F461.3B) was purchased from the Developmental Studies Hybridoma Bank. Mouse anti-nestin (catalogue number MAB353) and mouse anti-RNApol II (catalogue number 05-623, CTD4H8) were purchased from Millipore. Mouse anti-GAPDH (glyceraldehyde-3-phosphate dehydrogenase) (GTX627408) was purchased from Gene Tex. Anti-actin monoclonal antibodies were provided by Professor Manuel Hernández, Cinvestav-IPN.

### Plasmids

The reporter vectors to test NICD/RBPJκ activity (wild-type and mutated versions, p4xwtCBF1Luc and p4xmtCBF1Luc respectively) were kindly donated by Professor Hayward. Sequences for RBPJκ elements are as follows: wtRBPJκ, 5′-GATCTGGTGTAAACACGCCGTGGGAAAAAATTTATG-3′; and mtRBPJκ, 5′-GATCTGGTGTAAACACGGGCTTGGAAAAAATTTATG-3′(Hsieh and Hayward, 1995). pFCDN1, a vector expressing NICD, was kindly donated by Professor Gabriel Corfas (Patten et al., 2003). pGF1L luciferase reporter vector that contains the mouse GFAP promoter was kindly donated by Professor Nakashima (Asano et al., 2009). The CRE-CAT reporter vector contains the structural gene for CAT (chloramphenicol acetyltransferase), under the control of the HSV-TK (Herpes virus thymidine kinase) promoter and five CRE (cAMP-response element) elements (Rutberg et al., 1999).

### Cell culture and treatments

C6 cells were grown in DMEM (Dulbecco's modified Eagle's medium, Sigma–Aldrich) containing 5% FBS (PAA Laboratories), 120 units/ml penicillin (Penprocilin 800000 units, Lakeside), 150 μg/ml streptomycin (Sulfestrep, Pisa Laboratories) and 40 μg/ml gentamicin (Schering Plough), at 37°C under 5% CO_2_. When cells were at 80% confluence, treatments were applied in DMEM/0.5% FBS. Unless otherwise indicated, dbcAMP was applied to a final concentration of 750 μM and replaced every 48 h. In all cases when DAPT and H89 were added before treatment, vehicle (DMSO) was also tested. The highest DMSO concentration in media was 0.16% with no effect in control experiments (values were always similar to the untreated control).

### Transfection and reporter gene assays

Cells were seeded into 24-well plates (6.5×10^4^ cells/well) and transfected with 0.5μg of 4xwtCBF1Luc/4xmtCBF1Luc or 0.5μg of pGF1L constructs using Lipofectamine™ 2000 (GE Healthcare) in accordance with manufacturer's instructions. After 24 h, transfection media was removed and replaced with fresh culture medium. Cells were treated with dbcAMP or forskolin for 24 h. Inhibitors (H89 and DAPT) were added 30 min before treatment. Luciferase activity was determined using the Luciferase Assay System (Promega); and activity values were normalized to protein content and expressed relative to those recorded in non-treated controls.

For pCRE-CAT, 1.3×10^5^ cells/well were cultured on to 12-well plates and transfected with 2.1 μg of the vector using Lipofectamine™ 2000 as described above. After 24 h, transfection media was removed and replaced with fresh culture medium; and the culture treated with dbcAMP and forskolin. Protein lysates were obtained using 5×reporter lysis buffer (Promega). Equal amounts of protein lysates (~80 μg) were incubated with 0.25 μCi of [^14^C]chloramphenicol (50 mCi/mmol, Amersham Biosciences) and 0.8 mM acetyl-CoA (Sigma–Aldrich) at 37°C. Acetylated forms were separated by TLC and quantified using a Typhoon Optical Scanner (Molecular Dynamics). CAT activities were expressed as relative activities to non-treated control cell lysates.

For co-transfection experiments 1.25×10^6^ cells, 1.25 μg of p4xwtCBF1Luc/p4xmtCBF1Luc and 3.75 μg of pEF-BOS or pFCDN1 were used to perform the electroporation assay (200 mV, BTX Electroporator, Harvard Apparatus) (Neumann et al., 1982). After electroporation, cells were cultured for 48 h in DMEM/5% FBS. Luciferase activity was determined as described above.

### RT–qPCR (reverse transcription–quantitative PCR)

For RT-qPCR, C6 cells (6.5–7.5×10^5^ cells/well) were seeded into six-well plates. Cells were treated with dbcAMP (750 μM) and, when indicated, co-treated with DAPT (40 μM) or H89 (10 μM). Total RNA was extracted from cells using the Trizol® method (GE Healthcare). One microgram of total RNA was used in each assay. RT reactions were performed using the reverse transcriptase Improm-II (Promega), 0.1 μg of oligo(dT)_23_ as the primer (Fermentas) and 10 mM dNTPs [dATP, dGTP, dCTP, dTTP (GE Healthcare)] in a final volume of 20 μl with nuclease-free water (Promega). The reverse transcription step was performed at 25°C for 5 min, 37°C for 60 min and 70°C for 15 min. Quantitative real-time PCR was performed with SYBR green Ready Taq PCR Reaction Mix (Fermentas) for *GFAP*, *S100β*, *GLAST*, *PEN-2*, *Hes5*, *Notch1* and *GAPDH.* In all cases, the conditions were: after an initial cycle of 10 min at 94°C, 40 cycles of amplification (30 s at 94°C; 1 min at 60°C; 30 s at 72°C) and a melt curve (15 s at 95°C; 1 min at 60°C; 15 s at 95°C). Triplicate samples were subjected to qPCR using the Step One plus Real Time PCR System (Applied Biosystems). PCR amplifications were analyzed with Step one plus software (Applied Biosystems). The relative abundance of each mRNA is expressed as sample versus a control in comparison with *GAPDH* mRNA and was calculated using the 2^−ΔΔCt^ method. Primers used for amplification were as follows: *GFAP* sense, 5′-CCAAACTGGCTGACGTTTACC-3′; *GFAP* antisense, 5′-TGGTTTCATCTTGGAGCTTCTG-3′; *S100β* sense, 5′-GGTTG-CCCTCATTGATGTCT-3′; *S100β* antisense, 5′-CGTCTCCATC-ACTTTGTCCA-3′; *GLAST* sense, 5′-GGCTGCGGGCATTCCTC-3′; *GLAST* antisense, 5′-CGGAGACGATCCAAGAACCA-3′; *GAPDH* sense, 5′-GACCCCTTCATTGACCTCAAC-3′; *GAPDH* antisense, 5′-GTGGCAGTGATGGCATGGAC-3′; *Hes5* sense, 5′-GTGGT-GGAGAAGATGCGTCG-3′; *Hes5* antisense, 5′-GCTGTGTTTCAG-GTAGCTGACG-3′; *Notch1* sense, 5′-ATTTCACCGTGGGTGC-ACCG-3′; *Notch1* antisense, 5′-GTGTATCGGGCCCATCATGC-3′; *Pen-2* sense, 5′- TTGAACCTGTGCCGGAAGTA-3′; and *Pen-2* antisense, 5′- ATCACCCAGAAGAGGAAGCC-3′.

### Staining procedures

Cell culture staining with polyclonal and monoclonal antibodies was performed. C6 cells were grown in eight-well Lab-Tek Chamber Slides (Nalge Nunc International) with the same culture conditions and treatments as described above. Cells were fixed by exposure to acetone at −20°C for 3 min; and washed twice with 1×PBS. Cells were permeabilized with 1×PBS/0.25% Tween 20 (Bio-Rad Laboratories) and were blocked 30 min with IgG-free albumin (US Biological). Immediately, C6 cells were incubated with anti-GFAP (goat polyclonal, 1:50), anti-Notch1 (goat polyclonal, 1:50), anti-cleaved Notch1 (rabbit monoclonal, 1:50), anti-RNA pol II (mouse monoclonal, 1:100) or anti-Nestin (mouse monoclonal, 1:1000) antibodies for approximately 16 h at 4°C. The binding of the primary antibodies was visualized using fluorescein labeled anti-goat antibody (1:100, Invitrogen); Alexa Fluor 488 labeled anti-rabbit (1:200, Invitrogen); and Alexa Fluor 594 labeled anti-mouse (1:1000 or 1:400, Sigma–Aldrich and Invitrogen respectively). Control of immunolabeling was performed with the same staining procedure, using the visualizing reagents without the primary antibodies. Nuclei were counterstained using DAPI (dilution 1:1200; stock 2 mg/ml). The slides were mounted with Immu-mount (Thermo Scientific) and fluorescence was examined using a Leica confocal microscope. Coverslips were observed in a Leica TCS-SPE confocal microscope using an oil 63× objective (zoom 1; 1024×1024 pixel format). Images were obtained from exciting fluorochromes (wavelengths: 488 nm for FITC and Alexa Fluor 488; 594 nm for Alexa Fluor 594; and 358 nm for DAPI) for a single labeling. Co-localization ratio was determined using Leica LAS AF version 2.2.0, build 4758 software in 3D projections (stacked images) with a 30% background pixel and 50% threshold, equal for both channels. The co-localization ratio, as defined in Leica software (http://www.leica-microsystems.com) was determined selecting the area corresponding to the nuclei of each cell and using (as instructed by Leica) the formula:

$$\begin{array}{ccc} \mathrm{Co}-\mathrm{localization}\;\mathrm{Ratio} & = & \mathrm{Co}-\mathrm{localization}\;\mathrm{Area}/\mathrm{Area}\\ & & \mathrm{Foreground} \end{array}$$

AreaForeground=AreaImage−AreaBackground

### Protein extracts and subcellular fractionation

Whole-cell extracts from cultured C6 cells were prepared by scraping the cells in PBS buffer with protease and phosphatase inhibitors (1 mM PMSF, 10 mM Na_2_MoO_4_, 25 mM NaF, 1 mM Na_3_VO_4_). After centrifugation at 10015 ***g*** for 5 min, the cell pellet was lysed using the RIPA Lysis Buffer System (prepared in accordance with manufacturer's instructions, Santa Cruz Biotechnology) and vortex-mixed for 1 h at 4°C. Cell debris was discarded by centrifugation for 5 min at 15198 ***g***.

To isolate cytosolic and nuclear protein extracts, 5×10^6^ cells were pelleted and resuspended in 300 μl of TM-2 buffer [10 mM Tris/HCl, pH 7.4, 2 mM MgCl_2_, 0.5 mM PMSF, 1×Complete™ protease inhibitor (Roche Applied Science) and 2mM Na_2_VO_4_] and incubated for 10 min on ice; 300 μl of 5% (v/v) Triton X-100 was then added and the homogenate incubated on ice for 10 min. Cellular membrane debris was removed by 30 strokes in a Dounce homogenizer. Nuclei were separated from cytosol by centrifugation at 2432 ***g*** for 15 min at 4°C. The supernatant was saved as the cytosolic fraction and the pellet resuspended in 100 μl of sucrose buffer I [0.32 M sucrose, 10 mM Tris/HCl, pH 8, 3 mM CaCl_2_, 2 mM Mg(CH_3_COO)_2_, 0.1 mM EDTA, 1 mM DTT, 0.5 mM PMSF and 0.5% Nondet P40] and 100 μl of sucrose buffer II [2 M sucrose, 10 mM Tris/HCl, pH 8, 5 mM Mg(CH_3_COO)_2_, 0.1 mM EDTA, 1 mM DTT and 0.5 mM PMSF]. The nuclei suspension was transferred into a new tube with 200 μl of sucrose buffer II; after nuclei suspension, 1 ml of sucrose buffer I was added and then centrifuges at 16438 ***g*** for 180 min at 4°C (sucrose gradient formation). The nuclear pellet was suspended in lysis buffer (50 mM Tris/HCl, pH 8, 150 mM NaCl, 1% Triton X-100, 1 mM PMSF, 1×Complete™ protease inhibitor and 2 mM Na_2_VO_4_), sonicated and centrifuged at 16438 ***g*** for 2 min at 4°C, with supernatant retained as nuclear protein fraction.

### SDS/PAGE and Western blot analysis

For all extracts, a small aliquot was used for protein concentration determination by the Bradford method (Bradford, 1976). Equal amounts of protein (quantities are indicated in the Figure legends) were diluted in Laemmli's sample buffer, boiled for 10 min and analyzed through 6 or 10% polyacrylamide gel electrophoresis (SDS/PAGE). Proteins were transferred on to PVDF membranes and stained with Ponceau S solution to confirm equal loading of proteins. The blots were soaked in 1×TBS to remove Ponceau staining and incubated with the primary antibodies [anti-cleaved Notch1 (1:100), anti-Notch1 (1:1500), anti Hes5 (1:2000), anti-PEN2 (1:200), anti-actin (1:50) and anti-GAPDH (1:1000)] diluted in 0.25% BSA, 0.1% Tween 20 in 1×TBS buffer, followed by horseradish peroxidase-conjugated secondary antibodies. Finally, the protein bands were developed using an enhanced chemiluminescence kit (Amersham ECL Plus™ Western Blotting Detection Reagent) and exposed to X-ray films.

### Uptake assays

The uptake of [^3^H]D-aspartate used as a non-metabolizable analogue of glutamate, was performed as detailed elsewhere (Ruiz and Ortega, 1995). Briefly, the culture medium was exchanged with solution A (25 mM Hepes-Tris, 130 mM NaCl, 5.4 mM KCl, 1.8 mM CaCl_2_, 0.8 mM MgCl, 20 mM glucose and 1 mM NaHPO_4_, pH 7.4) and the cells were pre-incubated for 30 min at 37°C. Monolayers were incubated with solution A containing [^3^H]D-aspartate (0.4 μCi/ml) for 20 min. Thereafter, the medium was removed by rapid aspiration; the monolayers were washed with ice-cold solution A three times and solubilized with 0.1 M NaOH. Aliquots of the suspension were used for protein determination by the Bradford method (Bradford, 1976) and liquid scintillation counting. All of the uptake experiments were analyzed using GraphPad Prism software (see statistical analysis).

### Statistical analysis

Data are expressed as the means±S.E.M. A one-way ANOVA was performed to determine whether there were significant differences between conditions. When this analysis indicated significance (at the 0.05 level), post-hoc Student–Newman–Keuls test analysis was used to determine which conditions were significantly different from each other (Prism, GraphPad Software).

## RESULTS

### dbcAMP-PKA activation precedes NICD nuclear translocation in C6 cells

**Figure 1:**
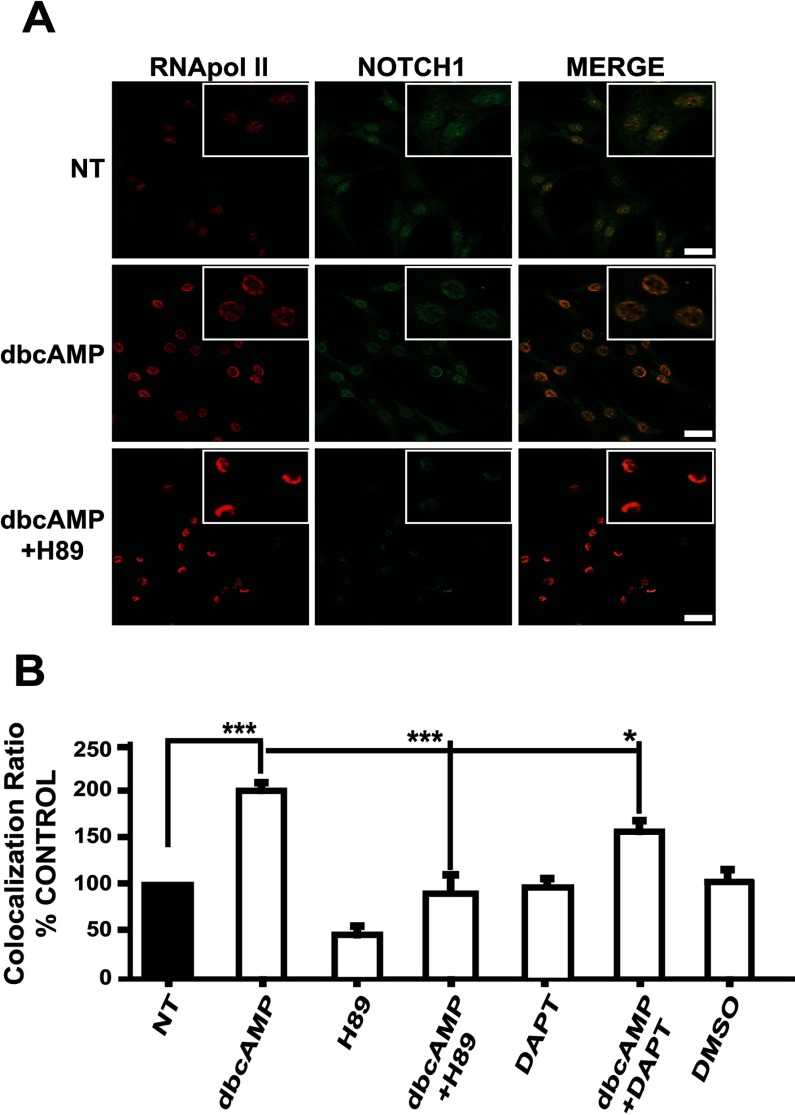
dbcAMP-PKA activation precedes NICD nuclear translocation in C6 cells C6 cells were treated with dbcAMP (750 μM); where indicated, the PKA inhibitor H89 (10 μM) or the γ-secretase inhibitor DAPT (40 μM) were added 30 min before dbcAMP; 24 h after treatment, cultures were processed for immunostaining. (**A**) Nuclear co-localization with anti-RNA pol II (red) and anti-Notch1 (C-terminal intracellular domain; green). (**B**) Co-localization ratios were determined with images (not shown) obtained with a Leica confocal microscope and the nuclei were counterstained with DAPI (blue) and using the software LAS AF version 2.2.0, build 4758 (Leica; scale bar=25 μm, as indicated in the Materials and methods section) to measure at least 30 cells per condition. Plotted data from at least three independent experiments are relative to the non-treated (NT) control; statistical analysis was performed using a non-parametric one-way ANOVA (Kurskal–Wallis test) and Dunn's post-hoc test (*P<0.05; ***P<0.001).

To analyze Notch pathway activity along the astrocytic differentiation induced by dbcAMP in C6 cells, NICD nuclear translocation was evaluated using several strategies. First, by immunostaining with an antibody directed against the C-terminal end of Notch1 that was used to locate the nuclear and cytoplasmic protein and to image its co-localization with a nuclear marker, RNA pol II. As depicted in Figure 1(A), after 24 h of dbcAMP exposure (750 μM), nuclear staining for Notch was increased (merged images). To test whether PKA activation leads to NICD nuclear translocation, we took advantage of the PKA inhibitor H89, noticing a clear blockage in nuclear translocation and also in the global Notch1 expression (Figure 1A).

Nuclear Notch signal was also quantified after treatment by recording co-localization ratios (see the Materials and methods section) for anti-Notch1-stained cells when nuclei were counterstained with DAPI. We did notice a 2-fold increase in the nuclear signal (histogram in 1B). In line with the results obtained in Figure 1(A), H89 blocks NICD nuclear translocation, meaning that the dbcAMP effect was completely abrogated. As expected, γ-secretase inhibition using DAPT also decreased NICD nuclear translocation by 50% in comparison with dbcAMP-treated cells (Figure 1B).

**Figure 2:**
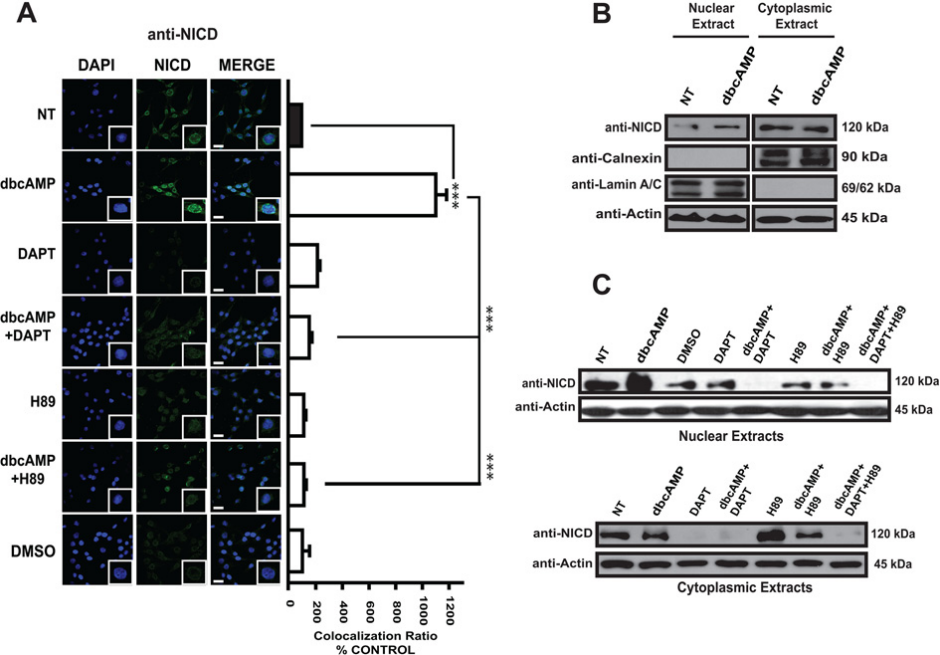
Only cleaved NICD is translocated from the cytoplasm to nuclei in a PKA- and γ-secretase-dependent manner In all cases, C6 cells were treated with dbcAMP (750 μM); where indicated, the γ-secretase inhibitor DAPT (40 μM) or the PKA inhibitor (H89) was added 30 min before dbcAMP treatment; 24 h after treatment, cells were processed. (**A**) Immunostaining for cleaved-NICD (Val^1744^; green); nuclei were counterstained with DAPI (blue). Images and co-localization ratios were obtained as in Figure 1 (scale bar=25 μm). (**B** and **C**) Subcellular fractionation and Western blot analysis using 75 μg of cytoplasmic or nuclear protein and anti-cleaved-NICD, anti-actin, anti-lamin A/C and anti-calnexin (as controls of nuclear and cytoplasmic extracts). Molecular masses are depicted on the right side. Representative images from at least three independent experiments are shown.

As a second approach, cleaved NICD was located (immunostained) using a specific antibody that recognizes Val^1744^, only detectable when NICD has been released by the action of γ-secretase. The cleaved form of Notch1 is located in the nuclei of C6 cells after 24 h of dbcAMP exposure (750 μM). Note that the same pathway (PKA and γ-secretase), is responsible for this localization (Figure 2A). The nuclear NICD observed in Figure 2(A), clearly comes from the specific cut induced after treatment, as Western blot analysis using nuclear extracts from dbcAMP-treated cells demonstrate an enhanced signal that matches with the decrease in the NICD cytoplasm detection (Figure 2B). Again, blockage of the PKA pathway avoids the appearance of nuclear NICD. Interestingly, a total disappearance from the nuclei is again clearly evident when both inhibitors are used, suggesting a cross-talk between PKA activation and the NICD cleavage that leads to NICD nuclear translocation. In this scenario, DAPT blocks all signal (Figure 2C).

### Cross-talk between PKA activation and NICD/RBPJκ-dependent transcription

**Figure 3:**
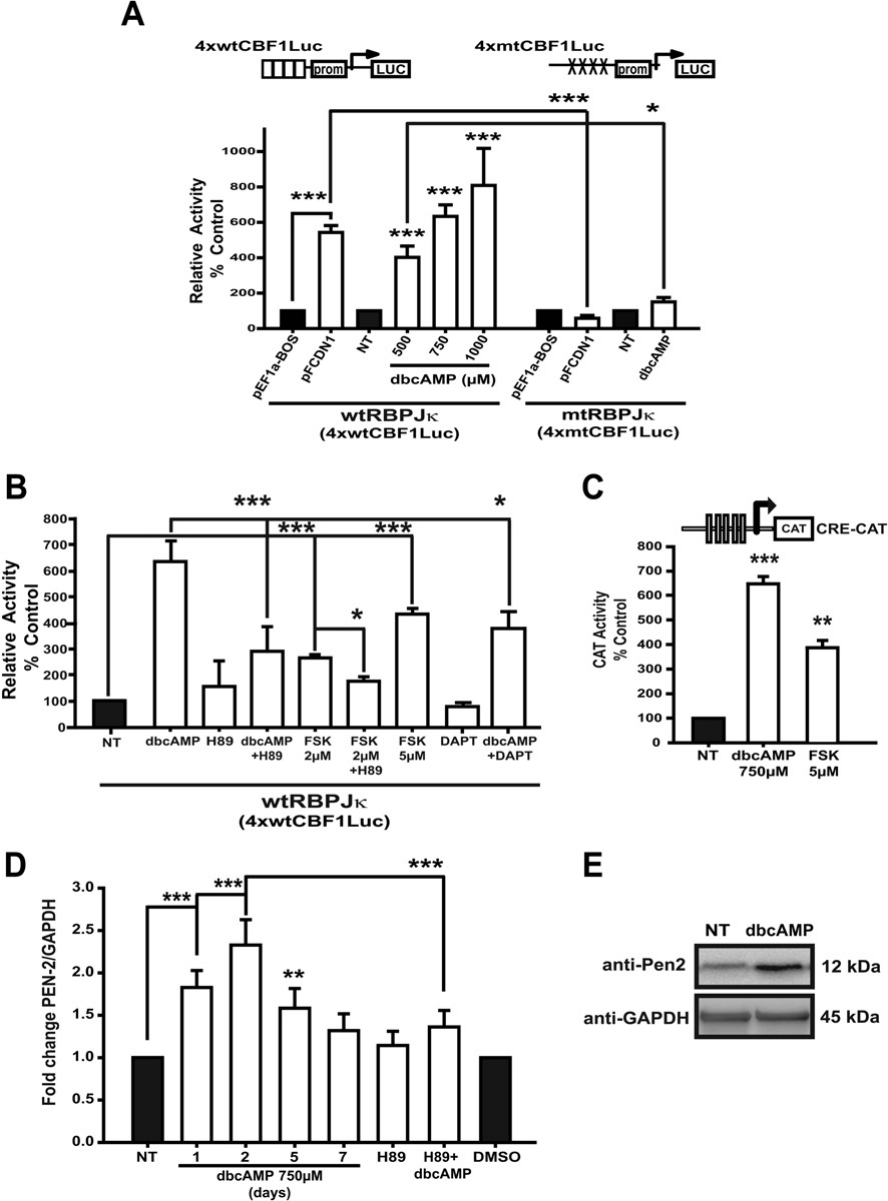
PKA-Notch pathway is involved in the NICD/RBPJk-dependent transcription during C6 astrocytic differentiation (**A**) C6 cells were transfected with 0.5 μg of the indicated reporter vectors for recording NICD/RBPJk-dependent transcription (4xwtCBF1Luc, wild-type/4xmtCBF1Luc, mutant version; schematic representations are shown in the upper part of the Figure); 24 h after transfection, cultures were treated with dbcAMP for another 24 h; when indicated, inhibitors were added as in Figure 1. Co-transfection assays were performed with 1.25 μg of 4xwtCBF1Luc or 4xmtCBF1Luc and 3.75 μg of NICD expressing vector (pFCDN1 and the pEF1a-BOS empty vector used as controls). (**B**) At 24 h after transfection with 0.5 μg of 4xwtCBF1Luc vector, cultures were treated with dbcAMP (750 μM) or forskolin (FSK, PKA inductor, indicated concentrations) for another 24 h; inhibitors were added 30 min before dbcAMP or FSK. (**C**) C6 cells were transfected with 2 μg of pCRE-CAT reporter construct, and 24 h after transfection treated with dbcAMP or FSK (as indicated) for another 24 h. In all cases, data are shown as relative to the activities in the non-treated (NT) control or the control co-transfected with the empty vector. (**D**) Total RNA was obtained from C6 cells treated as indicated and processed for qRT-PCR to amplify a 173 bp fragment from PEN-2 mRNA (nucleotides 143–315), using GAPDH levels as reference. Data are plotted relative to values recorded in non-treated (NT) and DMSO controls. (**E**) Approximately 100 μg of total protein extract from C6 cells treated with dbcAMP (750 μM, 2 days), were used for Western blot analysis with anti-PEN-2 and anti-GAPDH antibodies. Molecular masses are depicted on the right side. In all cases, data are plotted as means±S.E.M. from at least three independent experiments; statistical analysis was performed using a non-parametric one-way ANOVA (Kurskal–Wallis test) and Dunn's post-hoc test (*P<0.05; **P< 0.01; ***P<0.001).

NICD/RBPJκ-dependent transcription in dbcAMP-treated cells was determined using a reporter system directed by four RBPJκ-responsive elements (p4xwtCBF1Luc). As a positive control, C6 cells were co-transfected using the RBPJκ reporter plasmid plus the expression plasmid for NICD (pFCDN1). As expected, the 4xwtCBF1Luc vector activity, dependent on RBPJκ activation by NICD, showed a 6-fold increase in luciferase activity (Figure 3A). A progressive and significant (*P*<0.001) increase in NICD/RBPJκ-dependent transcription was observed in dbcAMP-treated cells; the maximal value was observed with 1 mM dbcAMP (Figure 3A). A mutated-reporter version (4xmtCBF1Luc vector, containing RBPJκ mutated elements) was used as an additional control. No increase in luciferase levels could be detected when C6 cells were transfected and treated or co-transfected with pEF1a-BOS-NICD (Figure 3A).

The increase in NICD/RBPJκ-dependent transcription observed with dbcAMP treatment was also severely impaired when PKA activation was blocked in cells transfected with the RBPJκ-responsive reporter plasmid and treated with dbcAMP plus H89 (Figure 3B). Forskolin, an established adenylate cyclase activator, augmented the NICD/RBPJκ-dependent transcription, but to a lesser extent (300–400%) than that observed with dbcAMP. Co-treatment with forskolin and H89 was used as a positive control. The increase in NICD/RBPJk-dependent transcription observed with dbcAMP was significantly decreased (40%) when γ-secretase was inhibited (*P*< 0.05) (Figure 3B). Additionally, PKA/CREB activation was evaluated with a CAT reporter plasmid directed by five CREB-responsive elements in dbcAMP-treated cells. The results shown in Figure 3(C) depict a 6-fold increase in CREB-dependent transcription (*P*<0.001).

A consequence of CREB activation is PEN-2 up-regulation, as was previously demonstrated (Wang et al., 2006). PEN-2 expression was determined measuring mRNA and protein levels under dbcAMP treatment. Under these conditions, PEN-2 protein and mRNA levels were increased, reaching maximum by the second day after treatment (Figures 3D and 3E). The latter effect on mRNA levels was blocked when the PKA inhibitor was added (Figure 3D). These results clearly demonstrate that during astrocytic C6 cell differentiation, a cAMP/PKA/Notch1 signaling cascade is taking place, and open the possibility that up-regulation of PEN-2, a key component of the γ-secretase complex, could be the connection between both pathways.

### The Notch1 target Hes5 is regulated by PKA/NICD/RBPJκ activation

**Figure 4:**
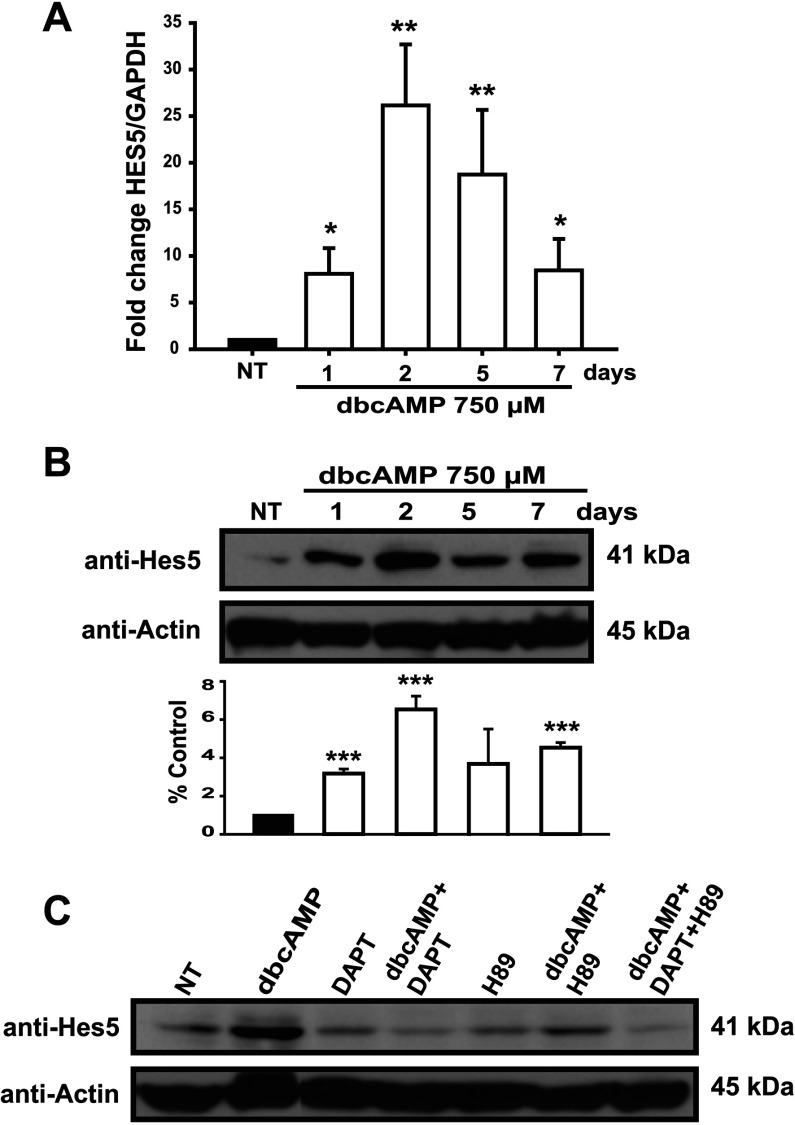
PKA/Notch activation induces Hes5 expression (**A**) Treated C6 cells were processed for qRT-PCR at the indicated times (days) to amplify a 150 bp fragment from Hes5 mRNA (nucleotides 119–268) and using GAPDH levels as reference. At each harvesting time, a non-treated matching control was also processed. (**B**) Cells were processed (at the indicated times) to obtain whole-cell extracts for Western blot analysis (approximately 30 μg) to identify Hes5 and actin; histogram shows densitometry data relative to the non-treated (NT) control and using actin as a reference. (**C**) Cells were pre-treated with H89 and DAPT (as in Figure 1) before dbcAMP stimuli; dbcAMP+DAPT+H89 stands for 750 μM, 20 μM and 5 μM, respectively. 48 h after treatment, total extracts were obtained and analyzed to detect Hes5 and actin by Western blot, non-treated controls were harvested at the final point (48 h). In (**B** and **C**), a representative Western blot is shown. Data are plotted as means±S.E.M. from three independent experiments expressed and statistically analyzed as in Figure 1 (*P<0.05; **P<0.01; ***P<0.001).

We next decided to evaluate Hes5 expression at mRNA and protein levels. A significant increase both in protein and mRNA levels was detected after the first day of dbcAMP treatment, reaching their maximal value (25-fold) and (7-fold) by the second day and progressively decreasing thereafter; although the basal levels were not recovered (Figures 4A and 4B). To demonstrate that indeed the dbcAMP effect on Hes5 expression was mediated though Notch1, cells were exposed to dbcAMP during 48 h in the presence of the γ-secretase inhibitor DAPT and a clear reduction in Hes5 was evident, and this effect is even more pronounced when the dbcAMP-treated cells were exposed to both DAPT and H89 (Figure 4C).

### PKA-Notch signaling is required for the astrocytic character: Notch involvement in astrocytic differentiation

**Figure 5:**
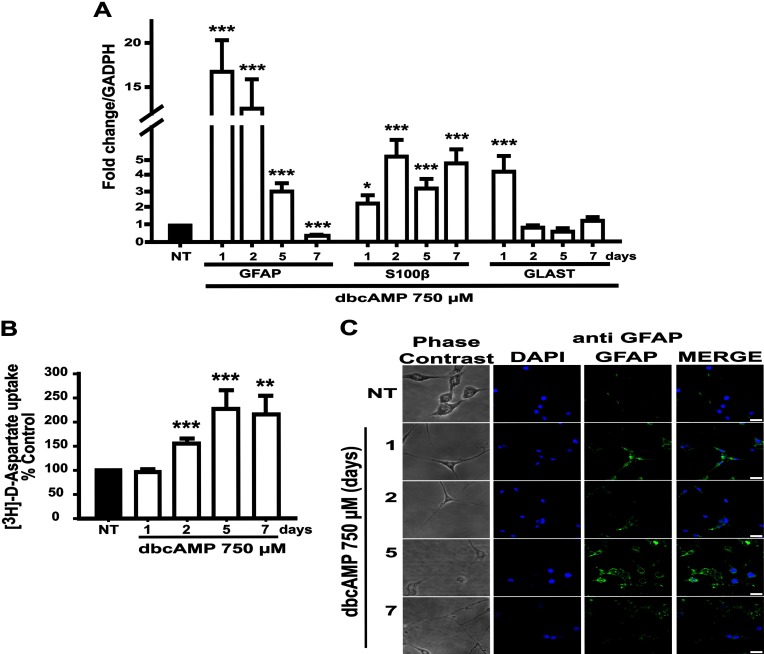
dbcAMP-induced astrocytic differentiation of C6 cells: glutamate transport and glial markers expression C6 cells treated as indicated and were subjected to [^3^H]D-aspartate uptake assays (performed in quadruplicates and normalizing data for each day/harvest with the respective non-treated control where cell viability was used to adjust uptake) in (**A**). In (**B**), total RNA was obtained from C6 cells treated as indicated and processed for qRT-PCR to amplify a 150 bp fragment from GFAP mRNA (nucleotides 324–473), a 157 bp fragment from S100β mRNA (nucleotides 144–300), a 120 bp fragment from GLAST mRNA (nucleotides 1520–1639), and using GAPDH levels as reference. mRNA levels were normalized for each day with data from the paired non-treated control, harvested in each point. Data are plotted as means±S.E.M. from at least three independent experiments and relative to values recorded in non-treated (NT) control harvested at the end of the treatment. Statistical analysis was performed using a non-parametric one-way ANOVA (Kurskal–Wallis test) and Dunn's post-hoc test (*P<0.05; **P<0.01; ***P<0.001). (**C**) C6 cells were treated with dbcAMP (750 μM, indicated times) and then immunostained with anti-GFAP (green). Nuclei were counterstained with DAPI (blue). Images were obtained using confocal and phase contrast microscopy (scale bar=25 μm).

Astrocytic differentiation upon dbcAMP treatment in C6 cells has been well established. In our hands, the differentiation markers *GFAP* and *S100β* mRNA levels also reached their maximum by the first and second day after treatment, respectively (Figure 5A). Interestingly, *GFAP* mRNA expression decreased during the following days of treatment, whereas *S100β* levels remained fairly stable. Nevertheless, the expression of both markers was always above basal levels shown by non-treated controls (followed and harvested at the same time/conditions in each case, see Figure legend). Similar to *GFAP* mRNA expression, *GLAST* mRNA showed an increase after the first day of treatment that gradually declined throughout the differentiation period. Accordingly, GLAST function detected through [^3^H]D-aspartate uptake was also increased (Figure 5B). Interestingly, while *GLAST* mRNA levels decreased after day 1, the [^3^H]D-aspartate uptake activity increased during the differentiation program (Figure 5B). Morphological changes were also present with dbcAMP (750 μM) treatment. A bipolar to stellate-like phenotype was observed from the first day of treatment. The cell body decreased gradually and reached its minimum at day seven; conversely, the length of cell elongations was also gradually augmented (Figure 5C). Additionally, a progressive disappearance of nestin, the intermediate filament marker for neural precursor cells was also noticed (data not shown).

**Figure 6:**
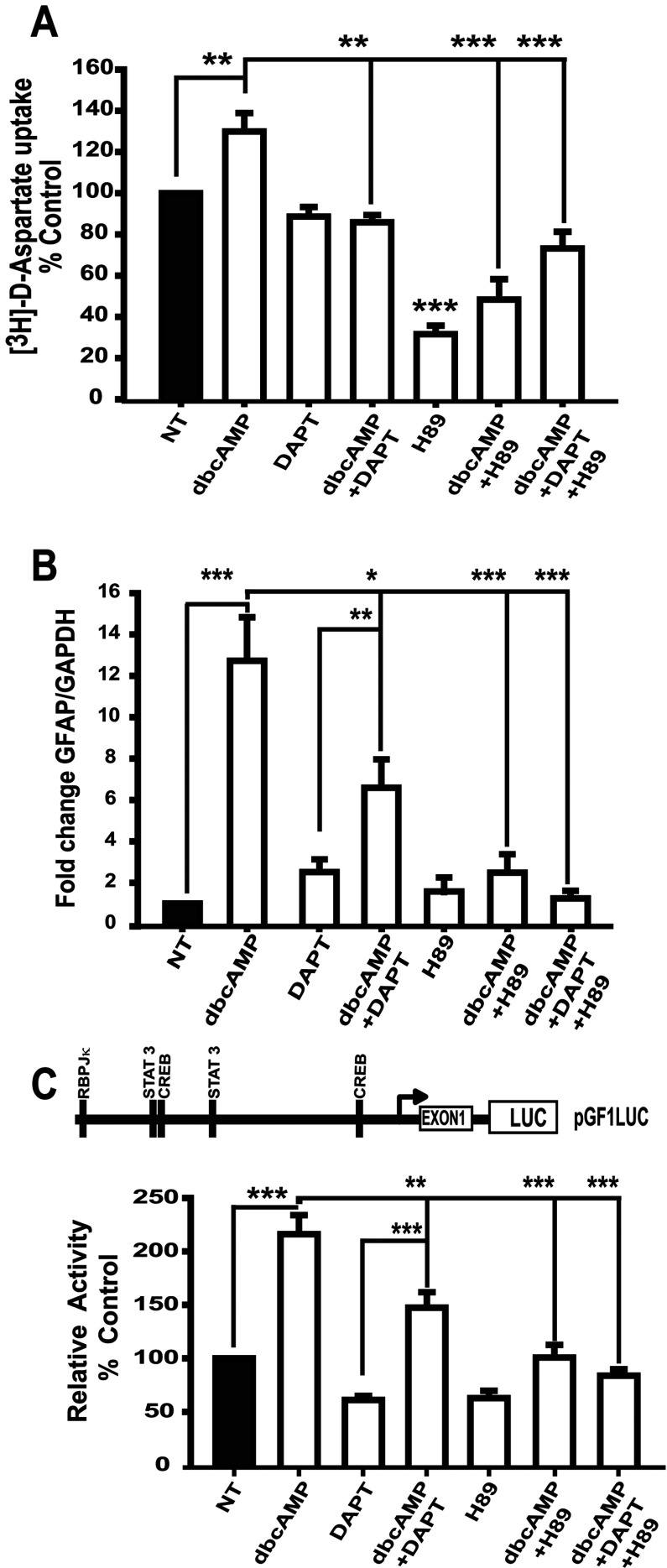
dbcAMP-PKA-Notch signaling involvement in astrocytic differentiation of C6 cells C6 cells were treated with dbcAMP (750 μM) and, when indicated, DAPT (40 μM) or H89 (10 μM) or DAPT/H89 (20 μM/5 μM) inhibitors were added 30 min before dbcAMP stimuli. (**A**) [^3^H]D-aspartate uptake assays, where cells were harvested 24 h after treatment. (**B**) qRT-PCR to amplify GFAP mRNA cells (harvested 24 h after treatment). (**C**) Map of pGLF1-Luciferase reporter for testing GFAP promoter-dependent transcription. Cells were transfected with 0.5 μg of pGF1L reporter and 24 h after treated with dbcAMP (750 μM, 24 h). When indicated, before dbcAMP stimuli (30 min), cells were treated as in (**A**). Data are shown as relative to values recorded in non-treated (NT) control and plotted as means±S.E.M. from at least three independent experiments. Statistical analysis was performed using a non-parametric one-way ANOVA (Kurskal–Wallis test) and Dunn's post-hoc test (*P<0.05; **P<0.01; ***P<0.001).

To confirm that PKA signaling is upstream of the Notch pathway signaling in astrocytic differentiation of C6 cells, GLAST function ([^3^H]D-aspartate uptake) and GFAP transcription were challenged with H89 and DAPT inhibitors and both were effectively blocked (Figure 6). We then decided to explore the effects of H89 and DAPT directly on the activity of the GFAP promoter. As expected, dbcAMP increased GFAP promoter transcription by 2-fold; however, this effect was diminished upon PKA or Notch receptor blockage by 50% and 100%, respectively, suggesting that PKA-mediated effects are partly dependent on Notch-1. To test this interpretation, cells were pre-exposed to DAPT and H89 at the same time. The results are shown in Figure 6(B), where a complete inhibition of the dbcAMP response was detected. These results suggest that GFAP is a target gene for the cAMP/PKA and Notch pathways, although Notch1 does not mediate all of the PKA effect.

### PKA-Notch signaling is required for Notch1 expression

**Figure 7:**
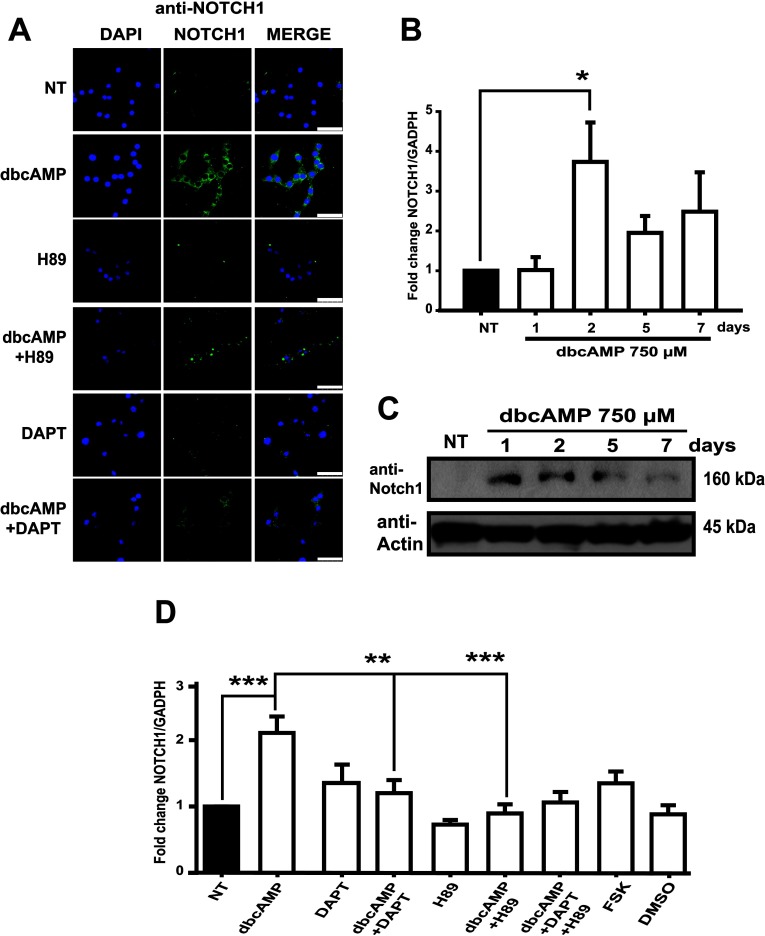
Induction (activation) of PKA-Notch pathway up-regulates Notch1 gene expression during astrocytic differentiation of C6 cells (**A**) Immunostaining and imaging obtained with a Leica confocal microscope and using anti-Notch1 (green) and the nuclei were counterstained with DAPI (blue) in C6 cells treated as indicated (the same conditions used in previous Figures, harvesting 48 h after treatment). Scale bar=50 μm. (**B**) Total RNA was obtained from C6 cells treated as indicated and processed for qRT-PCR to amplify a 170 bp fragment from Notch1 mRNA (nucleotides 7094–7263). (**C**) Approximately 30 μg of total protein extract from dbcAMP-treated cells were processed for Western blot to identify Notch1 using an antibody designed for detecting only the extracellular portion. (**D**) A representative Western blot is shown. qRT-PCR for Notch1 mRNA in C6 cells treated as indicated (the same conditions used in previous Figures, harvesting 48 h after treatment). For all qRT-PCR, data are plotted as means±S.E.M. from a minimum of three independent experiments and relative to values recorded in non-treated (NT) control and using GAPDH levels as reference. Statistical analysis was performed using a non-parametric one-way ANOVA (Kurskal–Wallis test) and Dunn's post-hoc test (**P<0.01; ***P<0.001).

Control, non-differentiated C6 cells present membrane and cytoplasmic Notch1, which increases after dbcAMP exposure (Figures 1A, 7A and 7B). *Notch1* mRNA levels are significantly increased after 2 days of dbcAMP treatment to progressively decrease in the following days, returning to almost basal levels at day seven (Figure 7B). Protein detection confirmed this observation (Figure 7C). Notch1 increase was abrogated when PKA activation or Notch receptor cleavage were inhibited. The inhibitory effect was also observed when a combination of DAPT and H89 was used (Figure 7D), pointing out that Notch1 receptor expression is under the control of the cross-talk between PKA and Notch pathways in the process of astrocytic differentiation in C6 cells.

## DISCUSSION

The molecular mechanisms involved in CNS differentiation programs have attracted the attention of an ever-increasing number of research teams. A fine tuning of the ratio of neurons to glia cells is needed for the proper development of the neuronal circuits (Chenn, 2009). An established role for PACAP in gliogenesis is well documented; under physiological conditions increased cAMP levels regulate GFAP gene expression (Tatsuno et al., 1996). Furthermore, it has been reported that PAC1 activation initiates morphological changes represented as process elongation in NPCs. Interestingly, in this system, the IP_3_ (inositol 1,4,5-triphosphate)/PKC (protein kinase C) pathway, rather than the cAMP/PKA cascade is involved in the induction of an astrocyte-like morphology (Nishimoto et al., 2007).

In the C6 cell model system, increasing cAMP levels activates PKA, stimulates IL-6 production, and consequently increases GFAP transcription via a PKA-CREB and JAK-STAT3 signaling cascade (Takanaga et al., 2004). Notch signaling is another key pathway implicated in astrogenesis (de la Pompa et al., 1997; Hitoshi et al., 2002; Yoon and Gaiano, 2005). With this in mind, we decided to test whether these two signaling systems overlap in C6 differentiation. To our surprise, we found that the non-hydrolysable cAMP analog, dbcAMP, triggers Notch signaling in a PKA-dependent manner. Although undifferentiated C6 cells express Notch ligands (data not shown), a barely detectable Notch activity is present in these cells independently of their confluence. In sharp contrast, dbcAMP exposure leads to an efficient NICD nuclear translocation and NICD/RBPJκ-dependent transcription. Thus far, NICD/RBPJk has been known to be activated only by the receptor–ligand interaction (Blaumueller et al., 1997). However, during vascular development, increased cAMP levels lead to Notch pathway activation to induce arterial endothelial cells from vascular progenitors (Yurugi-Kobayashi et al., 2006).

Our results suggest that C6 cells are useful for the study of astrocyte differentiation. The exposure of C6 cell monolayers to dbcAMP induces astrocytic morphological changes and the expression of the astrocytic markers GFAP, S100β and GLAST (Figure 5). A concomitant increase in [^3^H]D-aspartate uptake and a down-regulation of nestin expression (data not shown), an accepted marker for NSCs (neural stem cells) or glial progenitors (Mignone et al., 2004), confirms the role of dbcAMP in astrocyte differentiation (Tatsuno et al., 1996; Takanaga et al. 2004; Nakamachi et al., 2011). S100β and GFAP were also used as astrocytic markers because radial glia or adult NSCs do not express S100β, and oligodendrocytes do not express GFAP (Hachem et al. 2005; Cahoy et al. 2008). GFAP is also expressed by adult NSCs and radial glia (Patten et al. 2003; Liu et al. 2006), whereas S100β can be found in mature oligodendrocytes (Hachem et al., 2005).

GFAP mRNA levels are higher by day one and decreased through the following days. GFAP protein half-life is high (approximately 21 days) and only a pool of mRNA to produce GFAP protein and assembly filaments to form astrocytic shape seems to be necessary (Rolland et al., 1990). In contrast, *S100β* mRNA levels reached the maximum by the second day and keep at this level through the seven days. S100β has been implicated in the regulation of microtubule assembly of type III intermediate filaments (Brozzi et al., 2009; Raponi et al., 2007). Supporting this, Raponi et al. (2007) demonstrated that the S100β expression defines a late developmental stage after which GFAP-expressing cells (astrocytic precursors) lose their NSC potential. We also found an increased astrocytic function represented by [^3^H]D-aspartate uptake activity (Flott and Seifert, 1991), with an uptake increased since day two and reaching maximum at 5–7 days after treatment, and observed that *GLAST* mRNA increased by the first day after dbcAMP and dramatically decreases to basal levels over the next day of treatment. It seems that, similar to GFAP, only a pool of *GLAST* mRNA is necessary to accelerate the glutamate transporter. Other possibility is that GLT1 glutamate transporter in C6 cells (Baber and Haghighat, 2010) is contributing to the uptake.

It is important to mention that PKA activation was necessary for Notch pathway activation (Figures 2–4). At this stage, the obvious question of the mechanism involved in cAMP-Notch activation arose. In this context, it is known that Notch receptor cleavage is regulated by the γ-secretase complex, and that this activity is regulated by auto-proteolysis of presenilin (the catalytic subunit) initiated by PEN-2 assembly (Marlow et al., 2003; Bammens et al., 2011). Interestingly, PEN-2 regulation, and thus γ-secretase assembly, depends on the CREB transcription factor (Wang et al., 2006) providing a clue for the interpretation of the present results. During C6 exposure to dbcAMP, PEN-2 is transcriptionally up-regulated via CREB (Figure 3). PEN-2 might be the link between PKA and the Notch pathway, since this component is fundamental in presenilin activation in the γ-secretase complex (Takeo et al., 2012). Accordingly, *PEN-2* mRNA levels under dbcAMP treatment increase by the first day of treatment, reaching a maximum by day 2 (Figures 3D and 3E), therefore it is quite possible that PKA augments presinilin-1 levels in our system as has been shown to occur in neurons (Mitsuda et al. 2001a, 2001b).

During embryonic development, Hes1 is expressed in the early stages of neuroepithelial cell differentiation to prevent precocious neurogenesis. In contrast, Hes5 is expressed in the late stages, exactly when radial glia undergo the so-called astrocytic conversion (reviewed in Ohtsuka et al., 1999; Kageyama and Ohtsuka, 1999; Kageyama et al., 2008). Interestingly, in the C6 cell culture system, the cAMP-triggered Notch pathway results in Hes5, but not Hes1, expression (data not shown). Through the analysis of Hes5 expression, we confirmed that PKA is upstream of Notch activation and capable of regulating Hes5 expression, most likely to block pro-neuronal gene expression in glial progenitors.

The involvement of PKA in C6 astrocytic differentiation was demonstrated by its participation in the regulation of GFAP and Notch1 receptor genes; both directly, through CREB activation, and indirectly, through Notch receptor cleavage and then NICD/RBPJκ activation. Independent studies have demonstrated that CREB and NICD/RBPJκ regulate GFAP expression, Ge and co-workers have demonstrated that both STAT3 [activated by a LIF (leukemia inhibitory factor)-JAK stimulus] and NICD/RBPJκ are independent pathways that regulate GFAP expression (Ge et al., 2002). Our results demonstrate that the NICD/RBPJκ complex and CREB co-operate to regulate GFAP expression. It is quite possible that CREB and NICD/RBPJκ enhance transcription through the recruiting of chromatin-remodeling factors. A plausible explanation would be the recruitment of NICD by interaction with the phosphorylated form of CREB, and consequent binding of the co-activator p300 (HAT). Such a mechanism has indeed been described for the regulation of granzyme B expression in CD8^+^ T lymphocytes (Maekawa et al., 2008).

**Figure 8:**
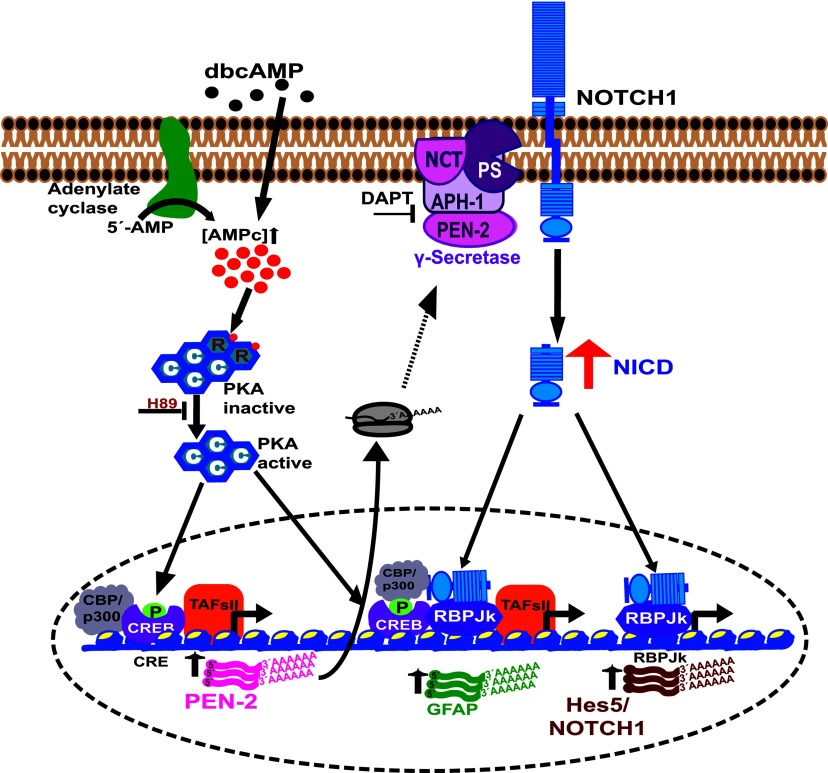
Current model cAMP/PKA/CREB and NICD/RBPJκ signaling regulate the expression of the astrocytic marker GFAP and the Notch pathway components (Notch1 receptor and Hes5 transcription factor) during astrocytic differentiation of C6 cells.

Considering that, once activated, the Notch1 receptor signals only once, it can be stated that this pathway strictly depends on receptor availability. In fact, the mechanisms of transcriptional regulation of the Notch1 receptor during neurogenesis and gliogenesis have been suggested (Aguirre et al., 2010; Cao et al., 2010). Therefore it is not surprising that in C6 cells, dbcAMP regulates Notch1 expression at the transcriptional level (Figure 7). Furthermore, the increase in Notch1 by PKA activity suggests the establishment of a positive-feedback loop that could complement our understanding of the neurogenesis/gliogenesis switch in the development of the CNS. A working model of our findings is depicted in Figure 8.
